# New CGRP Antagonists

**DOI:** 10.1100/tsw.2001.410

**Published:** 2001-12-12

**Authors:** H. N. Doods, M. Schindler

**Affiliations:** Department of Cardiovascular Research, Boehringer Ingelheim Pharma KG, Biberach, Germany

## Abstract

The presentation will focus on BIBN 4096 BS, since this compound is still the only potent calcitonin gene-related peptide (CGRP)-antagonist described so far[1].

